# Effects of legal and illegal use of benzodiazepines at acute admission to a psychiatric acute department

**DOI:** 10.1186/1756-0500-3-263

**Published:** 2010-10-19

**Authors:** John C Fløvig, Arne E Vaaler, Gunnar Morken

**Affiliations:** 1 Department of Neuroscience, Norwegian University of Science and Technology (NTNU), Trondheim, Norway; 2 Division of Psychiatry, Department Østmarka, St. Olav's University Hospital, Trondheim, Norway

## Abstract

**Background:**

In the psychiatric acute and emergency services patients present in severe crisis often complicated by behavioral problems, substance use, and multiple axis 1 diagnoses. In these clinical settings both legal and illegal use of benzodiazepines are difficult to evaluate since benzodiazepines could in some patients be regarded as first line treatment and in other patients as the cause of the acute psychiatric condition. The aims of this study were to evaluate the frequency and clinical effects of both legal and illegal use of benzodiazepines at admittance to a psychiatric acute department.

**Methods:**

All patients acutely admitted to a Norwegian acute psychiatric university department serving a catchment area were asked about use of benzodiazepines, other medications and substances before admission. Patients were asked to give urine samples for analyses of benzodiazepines and substances.

**Results:**

In 227 consecutive admissions there was legal use of benzodiazepines before admission in 39%, illegal use in 13% and no use in 48%. Patients with legal use of benzodiazepines were older, used more often antidepressants and a higher number of prescribed psychotropic medications. Illegal users of benzodiazepines more often used other illegal substances, were evaluated as clinically affected by a substance at admittance and were diagnosed with a substance use disorder. Patients with psychoses or major affective disorders treated with adequate medication (antidepressants, antipsychotics or mood-stabilizers) before admission more often received benzodiazepines than patients without adequate medication.

**Conclusions:**

The patients using benzodiazepines at admittance to psychiatric acute departments could be divided in illegal and legal users. The illegal users were young, used illegal substances and were more often regarded clinically affected by substances at admittance. The legal users were older, did not use other substances and were not regarded as clinically affected by substances at admittance. Benzodiazepines were used as adjuvant therapy to specific pharmacological treatment with antidepressants, antipsychotics or mood stabilizers for major psychiatric disorders.

**Trial registration:**

NCT 00184119/NCT 00184132

## Background

Use and abuse of benzodiazepines are difficult to evaluate in psychiatric acute admissions. Patients present in severe crisis often complicated by behavioral problems, substance use, and multiple axis 1 diagnoses [1]. In these clinical settings benzodiazepines are regarded as first line nonspecific treatment in patients suffering from acute agitated and psychotic conditions without known cause [2], and specific treatment in patients suffering from acute catatonia [3]. In patients suffering from severe agitation and substance abuse, benzodiazepines may also be regarded as first line acute treatment [4]. A substantial number of other patients suffering from psychoses or major depressive disorders use benzodiazepines as adjuvant symptomatic treatment to antipsychotics, mood stabilizers or antidepressants [2,5,6]. Patients with depression may however receive benzodiazepines for years without being treated with antidepressants [7-9].

Medication with benzodiazepines may have short and long time negative consequences. Frequently patients are admitted to psychiatric acute departments due to abstinence, intoxication, delirium and seizures [10]. Benzodiazepines are also abused in combination with illegal substances. Such abuse as part of a major psychiatric disorder is probably frequent [7].

Benzodiazepine use has been found in 40 to 45% of patients admitted to psychiatric hospitals [11-15], and being female and old increase the use of benzodiazepines [16,17]. Studies of illegal use of benzodiazepines in acute psychiatric admissions are few and the authors found no studies comparing legal and illegal use at admittance.

The aims of the present study were to assess the frequency of use of legal and illegal benzodiazepines at admissions to an acute psychiatric department and to compare patients using legal, illegal and no benzodiazepines.

## Methods

### Setting

The psychiatric acute department at Østmarka, St. Olav's University Hospital Trondheim, Norway had a catchment area of 140.000 inhabitants. About 700 patients above 18 years with acute psychiatric conditions were admitted each year. All patients in the catchment area suffering from acute psychiatric conditions with symptoms to a degree that locked in-patient ward was needed, were admitted to this department. Norwegian acute psychiatric services are public and available to everyone.

### Material

All acute admissions to inpatient treatment in a five month period were included. If patients were admitted more than once, all admissions were included.

At admission the patients were systematically asked about use of prescribed and non-prescribed psychotropic medication. All medications used before admission and still being prescribed at the time of admission were recorded. Benzodiazepine derivates sold for oral use in Norway were diazepam, oxazepam, alprazolam, clonazepam, flunitrazepam and nitrazepam. Zopiclone and zolpidem were categorized as non- benzodiazepine hypnotics. Use of benzodiazepines without a prescription is illegal in Norway [18].

The doctor on duty asked all patients at admission if they had used any substances during the last week. The doctor on duty also made an assessment whether the patient were clinically affected by substances or not.

All patients were asked to give urine samples as soon as possible and samples collected within 24 hours after admission were included in the study. Findings of substances used after admissions were not reported. The samples were analyzed with liquid chromatography with mass spectrometry (LC-MS). Substances in urine were reported as present or not present.

Use of substances or medications was defined by either the patient reported use of substances or medication during the last week before admission, the medication was reported used as a prescribed medication, or the substances or medication were detected in urine samples at admission. The following psychotropic medications were recorded: Antiepileptics, lithium, antidepressants, sedatives and anxiolytics, antipsychotics, psychostimulants, methadone and buprenorfin.

Diagnoses according to ICD-10 "Diagnostic criteria for research" [19] were set at discharge in a weekly consensus meeting in the department's staff including the patient's therapist and at least two psychiatrists of whom at least one personally knew the patient. In the present study both main and secondary diagnoses are reported.

The patients were assessed with the Global Assessment Scale Split version (GAF-S) at admittance [20]. GAF-S is based on DSM-4's GAF and is a two-item scale measuring global symptoms (GAF-S-Symptoms) and functioning (GAF-S-Function) separately [21].

In the group of patients with psychotic disorders (ICD-10 F 20.00 - F 29.99) use of antipsychotics in usual clinical doses for psychoses at admission was defined as adequate treatment. In patients with affective disorders (ICD-10 F 30.00 - F39.99) admitted with current episode depression, all antidepressants and mood stabilizing medication were defined as adequate. In addition atypical antipsychotics was defined as adequate treatment for patients with bipolar disorder, and all antipsychotics was defined as adequate treatment of mania [22].

The admissions were divided in legal use of benzodiazepines before admission defined as use of prescribed benzodiazepines, illegal use defined as use of non prescribed benzodiazepines or a combination of prescribed and non prescribed benzodiazepines, and no use of benzodiazepines before admission. Use of benzodiazepine was considered non-prescribed if there was no record of prescribed benzodiazepine for the last year before admission, or if a previous prescription was terminated by a doctor. New information received during the stay at hospital was included in the judgment. All written information from the admitting doctor and the hospital staff for the index admission and admissions from the last year before the index admission was reviewed in order to assure the right group assignment.

### Ethics

The study was approved by "The Regional Committee for Medical Research Ethics, Central Norway".

### Statistics

Categorical variables were analyzed using Chi- squared test or Fisher's exact test. Ordinal variables were compared using Mann-Whitney U-test/Kruskal Wallis test, and continuous variables were analyzed using Students t-test/ANOVA. Association of frequencies and age groups was analyzed with the Linear-by-linear association test. The α-level was set to 0.05. SSPS version 17.0 for Windows was used.

## Results

In the study period 184 patients with 227 admissions were included. Mean age was 39.5 years (SD 15.5 years). There were 95 women with 122 admissions and 89 men with 105 admissions.

There was legal use of benzodiazepines before admission in 89 (39%) of 227 admissions, illegal use in 30 (13%) admissions, 9 of these combined legal and illegal benzodiazepines, and no use in 108 (48%) admissions. Patients with legal use of benzodiazepines were older, used more often antidepressants and a higher number of prescribed psychotropic medications than the rest of the patients (Table 1).

**Table 1:** Use of psychotropic medications for patients using legal, illegal and no benzodiazepines.

	Legal (n = 89)	Illegal (n = 30)	No (n = 108)	All (n = 227)	P
Demographics					

Women^1 ^(%)	56 (63)	16 (53)	50 (46)	122 (54)	Ns

Age^2^	45.1	35.5	36.0	39.5	<0.001

Use of medications					

Antipsychotics^1 ^(%)	31 (35)	4 (13)	31 (29)	66 (29)	Ns

Antidepressants^1,3 ^(%)	34 (38)	10 (33)	23 (21)	67 (30)	0.031

Mood stabilizers^1 ^(%)	12 (13)	4 (13)	9 (8)	25 (11)	Ns

Number of prescribed psychotropic medications, not including benzodiazepines^4^	1.58	1.17	0.96	1.23	<0.001

^1^Chi-square test with 95% confidence intervals computed for each square if Chi-square test is significant.

^2^One-Way ANOVA test with Post Hoc Tamhane's tests, legal users were older than no users (P < 0.001) and older than illegal users (P = 0.004).

^3^No users of benzodiazepines used less antidepressants than legal users.

^4^Kruskal-Wallis tests with Post Hoc Mann- Whitney U tests, legal users used more other psychiatric medications than no users (p < 0.001).

In 65 of 104 (62.5%) admissions with psychoses or major affective disorders (ICD-10 F 20.00 - 39.99), the patients received adequate medication with antipsychotics, antidepressants or mood stabilizers before admission. Among the 65 patients receiving adequate medication, 34 (52%) used benzodiazepines while among the 39 patients that did not receive adequate medication 11 (28%) used benzodiazepines. (χ^2 ^= 5.77, df = 1, p = 0.016).

Legal users had a higher number of prescribed psychotropic medications not including benzodiazepines than no users (Table 1). Patients with psychoses or major affective disorders (ICD-10 F 20.00-39.00) treated with adequate medication, received a higher number of psychotropic medications not including benzodiazepines than patients without adequate medication (mean 1.91 (SD 0.861) vs. 0.33 (SD 0.621), p < 0.001) (Mann-Whitney U Test).

Illegal users of benzodiazepines more often used illegal opiates, cannabis and illegal psychostimulants, and were often evaluated as clinically affected by a substance at admittance (Table 2). The illegal users were also more often diagnosed with a substance use disorder (15/30 (50%) vs. 57/197 (29%), χ^2 ^= 5.33, df = 1, p = 0.02, a benzodiazepine use disorder (10/30 (33%) vs. 8/197 (4%) (Fisher's Exact test P < 0.001) and with a substance use disorder as the main diagnosis (10/30 (33%) vs. 16/197 (8%), χ^2 ^= 16.3, df = 1 p < 0.001). The frequency of benzodiazepine use disorders in legal users of benzodiazepine was 9% (8/89).

**Table 2:** Reported use of substances, doctor's evaluation and findings in urine samples^1 ^at admission.

	Legal (n = 89)	Illegal (n = 30)	No (n = 108)	All (n = 227)	P
Patient report					

Use of alcohol last week^2 ^(%)	29 (33)	15 (50)	49 (45)	93 (41)	Ns

Use of illegal opiates last week^3 ^(%)	3 (3)	**7 (23)**	0 (0)	10 (4)	<0.001

Use of cannabis last week^2 ^(%)	2 (2)	**9 (30)**	8 (7)	19 (8)	<0.001

Use of illegal stimulants last week^3 ^(%)	1 (1)	5 (17)	4 (4)	10 (4)	0.004

Doctor's assessment					

Patient regarded clinically affected by a substance^3 ^(%)	17 (19)	**11 (37)**	*7 (6)*	35 (15)	<0.001

Urine samples^2^	n = 75	n = 30	n = 91	n = 196	Ns

Ethanol in urine^3 ^(%)	4 (4)	2 (7)	5 (5)	11 (5)	Ns

Illegal opiates in urine^3 ^(%)	3 (3)	**5 (17)**	2 (2)	10 (4)	0.015

Cannabis in urine^2 ^(%)	6 (7)	**7 (23)**	7 (6)	20 (9)	0.036

Illegal stimulants in urine^3 ^(%)	0 (0)	5 (17)	4 (4)	9 (4)	0.001

Two or more benzo diazepines in urine sample^2 ^(%)	*9 (10)*	**10 (33)**	-	19 (8)	<0.001

Values **higher **or lower than expected are marked with bold or underlined italics, respectively.

^1^Urine samples analyzed with the LC-MS method.

^2^Chi-square test with 95% confidence interval computed for each square if Chi-square test is significant.

^3^Fisher's Exact test with 95% confidence interval computed for each square if Fisher's Exact test is significant.

Urine samples were given in 196 of 227 admissions (86%). The results are summarized in Table 2. The correlations between verbal reports of the patients and urine analyses are published previously [13]. In the benzodiazepine group 4 patients (3%) had denied use but tested positive.

Average score for GAF for all admissions was 38.2 for symptoms and 39.0 for function, with no differences between legal, illegal and no users of benzodiazepines.

**Figure 1:**
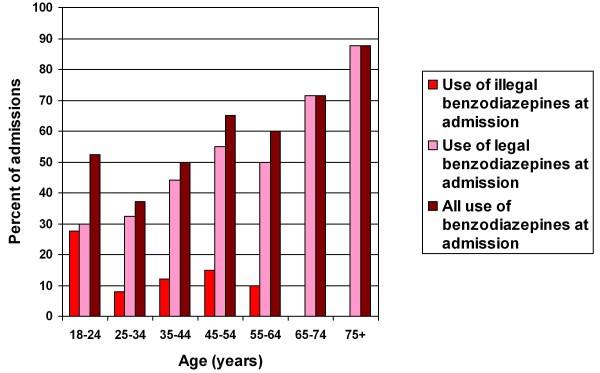
**Use of legal and illegal benzodiazepines at admission for 227 admissions to an acute psychiatric department**.

Legal use increased with age (Linear-by-linear association test, P < 0.001), and 80% of patients 65 years and older used benzodiazepines at admittance. Illegal use was most frequent in young patients (Linear-by-linear association test, P = 0.047) (Figure 1).

A somatic disease had been diagnosed in 73 admissions (32%). These were diseases of the nervous system (ICD-10 G00-G99) in 28 admissions, diseases of the circulatory system (I00-I99) in 25, diseases of the endocrine and metabolic system (E00-E90) in 17, diseases of the digestive system (K00-K93) in 6, diseases of the respiratory system (J00-J99) in 5 and various diseases in 18 admissions.

## Discussion

Patients admitted to psychiatric acute departments using benzodiazepines could be divided in two main groups. Illegal users were young, reported use of multiple illegal substances and were regarded as clinically affected by substances at admittance by the doctor on duty. Legal users were older, did not use multiple substances and were not regarded as clinically affected by substances at admittance. Among the illegal users, more than one benzodiazepine was frequent and the patients often had a substance use disorder. The findings at admittance were supported by the results from the laboratory tests.

In 62.5% of admissions with psychoses or major affective disorders the patients had received adequate medications before admission. Adequate medications were combined with benzodiazepines in 34 of 65 admissions while only 11 of 39 patients without adequate medication prior to admission received benzodiazepines. This indicates that patients with psychoses or affective disorders in risk of being admitted to psychiatric emergency departments are treated with benzodiazepines to relieve acute symptoms as long as the patients display symptoms sufficient for their psychiatric conditions to be diagnosed [2].

The major clinical challenge emerging from the present study is the relatively large population not receiving neither specific nor nonspecific pharmacological treatments before admission. This indicates that many acute major psychiatric episodes were neither recognized nor diagnosed before the acute emergency situation brought the patients to the acute department.

The use of antidepressants was lower among no users than among legal users of benzodiazepines (Table 1). We can not exclude the possibility that antidepressants induce adverse symptoms as agitation in sensitive individuals leading to more use of benzodiazepines [23,24]. Agitation is common in severe depression (40 - 70%) [23] and is a frequent cause of admittance to psychiatric emergency facilities [25]. Benzodiazepines are effective and safe in the treatment of agitation [26]. Our data therefore supports that use of benzodiazepines in our present acute population could be clinically well-funded.

The doctors were able by the clinical examination at admittance to indicate which patients were abusing benzodiazepines, and which patients were using benzodiazepines as a nonspecific, specific or adjuvant medication as part of the treatment of a major psychiatric disorder. The legal users were less often regarded as intoxicated than the illegal users. When using benzodiazepines as adjuvant treatment for major psychiatric disorders the need for such treatment would decline as specific treatment with antipsychotics, antidepressants or mood stabilizers have effect on the main disorder. The probability of long time negative consequences of using benzodiazepines as part of an addiction habit is less among the legal than the illegal users.

This study has limitations. "Doctor's evaluations" reflects the opinion of the experienced clinicians. We do not have data on how many patients have long-term use of benzodiazepines.

The present study is strengthened by the prospective design. All patients from a defined catchment area in need of acute psychiatric services were included. Laboratory tests were taken shortly after admittance to the acute department.

## Conclusions

The patients admitted to psychiatric acute departments using benzodiazepines can be divided in two main groups. Illegal users were young, used multiple illegal substances and were often evaluated as clinically affected by substances at admittance. Legal users were older, did not use illegal substances and were less frequently evaluated as clinically affected by substances at admittance. Benzodiazepines were often used as adjuvants to specific pharmacological treatment with antidepressants, antipsychotics or mood stabilizers for major psychiatric disorders.

## Competing interests

Dr. Fløvig has been a speaker in meetings arranged by Astra Zeneca, Janssen-Cilag and Lundbeck. Dr Morken has received a travel grant from Astra Zeneca. None of the authors have any financial interests or other potential conflicts of interest.

## Authors' contributions

JCF, AEV, and GM conceived and designed the study. JCF coordinated the study including the inclusion of patients. All authors helped to draft the manuscript, and all authors read and approved the final version.
